# Improving molecular property prediction through a task similarity enhanced transfer learning strategy

**DOI:** 10.1016/j.isci.2022.105231

**Published:** 2022-09-30

**Authors:** Han Li, Xinyi Zhao, Shuya Li, Fangping Wan, Dan Zhao, Jianyang Zeng

**Affiliations:** 1 Institute for Interdisciplinary Information Sciences, Tsinghua University, Beijing 100084, China; 2 Machine Biology Group, Departments of Psychiatry and Microbiology, Institute for Biomedical Informatics, Institute for Translational Medicine and Therapeutics, Perelman School of Medicine, Departments of Bioengineering and Chemical and Biomolecular Engineering, School of Engineering and Applied Science, Penn Institute for Computational Science, University of Pennsylvania, Philadelphia, PA 19104, USA

**Keywords:** Drugs, Computational chemistry, Bioinformatics, Artificial intelligence

## Abstract

Deeply understanding the properties (e.g., chemical or biological characteristics) of small molecules plays an essential role in drug development. A large number of molecular property datasets have been rapidly accumulated in recent years. However, most of these datasets contain only a limited amount of data, which hinders deep learning methods from making accurate predictions of the corresponding molecular properties. In this work, we propose a transfer learning strategy to alleviate such a data scarcity problem by exploiting the similarity between molecular property prediction tasks. We introduce an effective and interpretable computational framework, named MoTSE (Molecular Tasks Similarity Estimator), to provide an accurate estimation of task similarity. Comprehensive tests demonstrated that the task similarity derived from MoTSE can serve as useful guidance to improve the prediction performance of transfer learning on molecular properties. We also showed that MoTSE can capture the intrinsic relationships between molecular properties and provide meaningful interpretability for the derived similarity.

## Highlights

• MoTSE accurately measures similarity between molecular property prediction tasks
• A novel transfer learning strategy to accurately predict molecular properties
• An interpretable method to help understand relations between molecular properties

## Graphical abstract

**Figure:**
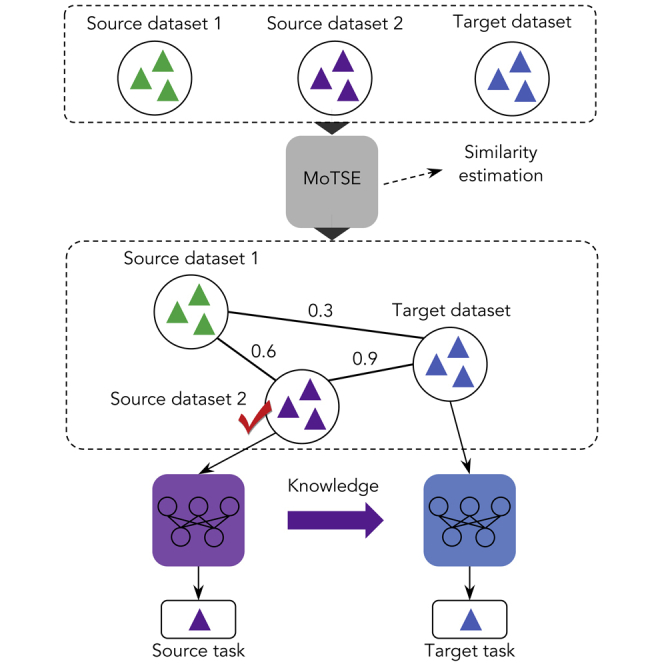

## Introduction

With the development of high-throughput experimental techniques in the fields of biology and chemistry (Macarron et al., 2011), the number of available datasets of diverse molecular properties has increased significantly over the past few years (Ramakrishnan et al., 2014; Papadatos et al., 2015; Kim et al., 2016). This offers an unprecedented opportunity to design accurate computational models for molecular property prediction, thus facilitating the comprehension of molecular properties and accelerating the drug discovery process. However, as huge experimental efforts are often required for obtaining large-scale molecular property labels, the available data of the majority of the properties are still extremely scarce. For example, although the preprocessed ChEMBL dataset (Gaulton et al., 2012; Mayr et al., 2018) contains 1,310 bioassays and covers over 400K small molecules, the numbers of available labels of over 90% of the bioassays are below 1K. This data scarcity problem has limited the applications of data-driven computational models, especially deep learning models, in making accurate predictions of the corresponding molecular properties.

To alleviate the data scarcity problem, transfer learning strategies have been widely applied to improve the prediction performance of tasks with limited data in the field of computer vision (Zamir et al., 2018; Li et al., 2020; Chen and He, 2021). The general idea of transfer learning strategies is to transfer the knowledge learned from a source task with sufficient data to enhance the learning of a target task with limited data. The superior performance of transfer learning has also been well validated in molecular property prediction tasks (Simoes et al., 2018; Shen and Nicolaou, 2020; Cai et al., 2020; Li and Fourches, 2020). Nevertheless, the success of transfer learning is not always guaranteed. A number of studies have indicated that transfer learning can harm prediction performance (termed negative transfer) (Rosenstein et al., 2005; Fang et al., 2015; Wang et al., 2019b; Zhuang et al., 2021). It has been observed that negative transfer usually occurs when there exists only weak (or even no) similarity between the source and target tasks (Zhang et al., 2020). Therefore, to facilitate the effective applications of transfer learning in molecular property prediction and avoid the negative transfer problem, it is necessary to accurately measure the similarity between different molecular property prediction tasks.

It is generally hard to explicitly and manually measure the similarity between molecular property prediction tasks, even for experienced experts, as fully understanding the behaviors of molecules in the chemical and biological systems is extremely difficult owing to the high complexity of these systems. Fortunately, data-driven computational methods can provide an implicit way to enable us to define and measure task similarity. The seminal work of Taskonomy (Zamir et al., 2018) has made a pioneering attempt toward modeling the similarity between computer vision tasks through a deep learning approach. The results have shown that incorporating the similarity derived from Taskonomy can improve the performance of transfer learning on computer vision tasks. In addition, the similarity tree constructed according to the derived similarity is highly consistent with human conceptions, indicating that such approaches can potentially capture the intrinsic relationships between tasks. This thus inspires us to develop a computational method for estimating the similarity between molecular property prediction tasks, which can not only guide the source task selection to avoid negative transfer in transfer learning but also provide useful hints in understanding the relationships between tasks.

To this end, we propose MoTSE, an interpretable computational framework, to efficiently measure the similarity between molecular property prediction tasks. MoTSE is based on the assumption that two tasks should be similar if the hidden knowledge learned by their task-specific models is close to each other. More specifically, MoTSE first pre-trains a graph neural network (GNN) model for each task. Then an attribution method and a molecular representation similarity analysis (MRSA) method are introduced to represent the hidden knowledge enclosed in the pre-trained GNNs as embedded vectors and project individual tasks into a unified latent space. Finally, MoTSE calculates the distances between the vectors in the latent space to derive the similarity between different tasks. Based on the task similarity derived from MoTSE, we design a novel transfer learning strategy to enhance the learning of the molecular property prediction tasks with limited data.

Our extensive computational tests demonstrated that the task similarity estimated by MoTSE can successfully guide the source task selection in transfer learning, with superior prediction performance over a number of baseline methods, including multitask learning, training from scratch, and nine state-of-the-art self-supervised learning methods, on several molecular property datasets from various domains. Meanwhile, by applying MoTSE to a dataset measuring the physical chemistry properties and a dataset measuring the bio-activities against cytochrome P450 isozymes, we also demonstrated that MoTSE was able to capture the intrinsic relationships between molecular properties and provide meaningful interpretability for the derived similarity.

## Results

### Overall design of MoTSE

**Figure 1:**
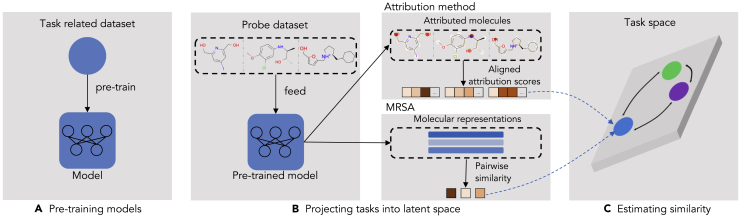
An illustrative diagram of MoTSE (A) Given a task, MoTSE first pre-trains a GNN model using the corresponding dataset in a supervised manner. (B) By means of a probe dataset, MoTSE extracts the task-related knowledge from the pre-trained GNN and projects the task into a latent task space. The knowledge extraction is achieved by two methods: an attribution method extracting the task-related local knowledge by assigning importance scores to atoms in molecules; and a molecular representation similarity analysis (MRSA) method extracting the task-related global knowledge by pair-wisely measuring the similarity between molecular representations. (C) Finally, MoTSE calculates the similarity between tasks by measuring the distances between the corresponding vectors in the task space.

Figure 1 illustrates the overall architecture of MoTSE. Given a set of molecular property prediction tasks with the corresponding datasets, MoTSE estimates the task similarity via the following three main steps: (1) Representing molecules as graphs, where nodes represent atoms and edges represent covalent bonds (see Figure S1), MoTSE pre-trains a graph neural network (GNN) model on the dataset for each task in a supervised manner. (2) By means of a probe dataset (i.e., a set of unlabeled molecules, see STAR Methods for more details), MoTSE extracts the task-related knowledge from the pre-trained GNNs and then projects the tasks into a unified latent task space. The knowledge extraction is achieved by an attribution method and a molecular representation similarity analysis (MRSA) method. These two methods are effectively complementary to each other: the attribution method extracts the local knowledge by assigning importance scores to atoms in molecules and the MRSA method extracts the global knowledge by pair-wisely measuring the similarity between molecular representations. (3) MoTSE estimates the similarity between tasks by calculating the distances between the corresponding vectors in the projected latent task space.

Based on the task similarity derived from MoTSE, we design a novel transfer learning strategy to improve the prediction performance for molecular properties with limited data. More specifically, given a target task, we first select the most similar task according to the task similarity estimated by MoTSE as its source task and then finetune the model pre-trained on the source task to exploit its related knowledge to enhance the learning of the target task. As GNN models have shown superior capability in learning hidden knowledge and modeling various kinds of molecular properties (Gilmer et al., 2017; Li et al., 2019; Xiong et al., 2019), here we also adopt the GNN models to capture the hidden knowledge contained in individual tasks. Note that, MoTSE is orthogonal to different GNN architectures. We use graph convolutional networks (GCNs) (Kipf and Welling, 2016) in our computational experiments if not specially specified (see Figure S2 for an illustrative diagram for our model architecture). More details about MoTSE, the transfer learning strategy, the model architecture, and the training process can be found in STAR Methods.

### The MoTSE-guided transfer learning strategy outperforms baseline methods

**Figure 2:**
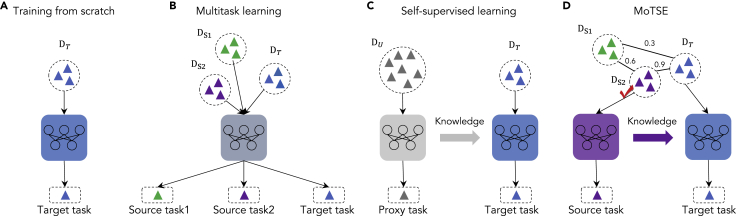
Schematic illustration of different learning strategies (A) Training from scratch directly trains a model on the dataset of each target task without exploiting any extra knowledge. (B) Multitask learning learns the target task and source tasks simultaneously. (C) Self-supervised learning first leverages a proxy task to learn general knowledge from a large-scale unlabeled dataset and then finetunes the pre-trained model on the dataset of the target task. (D) MoTSE-guided transfer learning first pre-trains a model on the most similar task with the target task according to the task similarity estimated by MoTSE and then finetuned the pre-trained model on the dataset of the target task. $D_{T}$ stands for the dataset for the target task, $D_{S1}$ and $D_{S2}$ stand for the datasets for the source tasks, and $D_{U}$ stands for the large-scale unlabeled dataset. The numbers between the datasets represent the similarity estimated by MoTSE between the corresponding tasks.

We systematically evaluated the performance of our MoTSE-guided transfer learning strategy on molecular property prediction. We made comparison with eleven baseline methods with different learning strategies, including multitask learning (MT), training from scratch (Scratch), and nine state-of-the-art self-supervised learning methods, i.e., EdgePred (Hamilton et al., 2017), DGI (Velickovic et al., 2019), Masking (Hu et al., 2020), ContextPred (Hu et al., 2020), JOAO (You et al., 2021), EdgePred_*sup*_ (Hu et al., 2020), Masking_*sup*_ (Hu et al., 2020), ContextPred_*sup*_ (Hu et al., 2020) and DGI_*sup*_ (Hu et al., 2020) (see STAR Methods for more details about these baseline methods). A schematic illustration of our MoTSE-guided transfer learning strategy and other learning schemes is shown in Figure 2.

We first applied the following two representative datasets QM9 (Ramakrishnan et al., 2014) and PCBA (Ramsundar et al., 2015) for performance evaluation, in which the QM9 dataset measured the quantum chemical properties and the PCBA dataset measured the bio-activities of small molecules (see Table S1 and STAR Methods for more details about the datasets used in our tests). To evaluate the effectiveness of different learning strategies, we further preprocessed the datasets to (1) mimic a specific scenario of transfer learning, in which the data size of the source task was relatively larger than that of the target task, and (2) reduce the influence of other factors (e.g., data size) that might affect the performance of transfer learning and thus only focus on the effect of learning strategies themselves. In particular, we first created two subsets QM9_10*k*_ and PCBA_10*k*_ as the datasets for the source tasks, in which each task had about 10,000 data samples, and then randomly partitioned the datasets into training, validation, and test sets with a ratio of 8:1:1. Next, we constructed another two subsets QM9_1*k*_ and PCBA_1*k*_ as the datasets for the target tasks by: (1) constructing training and validation sets by sampling 800 and 100 data samples from the corresponding training and validation sets of QM9_10*k*_ and PCBA_10*k*_, respectively, to avoid data leakage in the transfer learning; and (2) sharing the test sets with QM9_10*k*_ and PCBA_10*k*_, respectively, for an accurate performance evaluation (see Figure S3 for an illustrative diagram of the dataset generation process).

**Figure 3:**
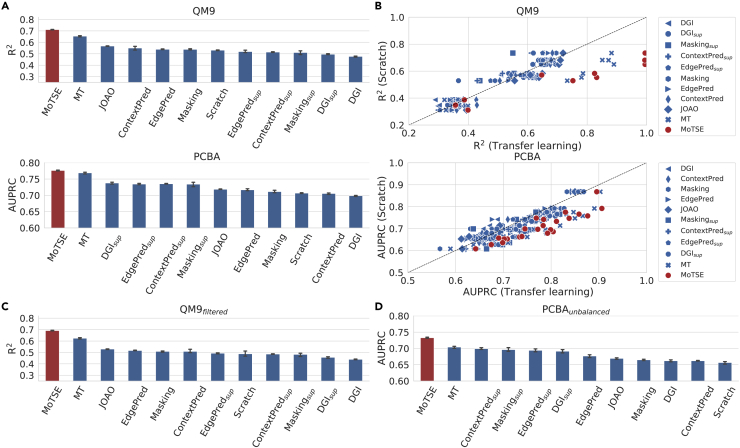
MoTSE outperforms baseline methods and alleviates negative transfer on the QM9 and PCBA datasets (A) The prediction performance of MoTSE and eleven baseline methods on the QM9 and PCBA datasets, measured in terms of R^2^ and AUPRC, respectively. (B) The prediction performance of eleven transfer learning methods versus that of the Scratch method on the QM9 and PCBA datasets. (C) The comparison results of R^2^ between MoTSE and eleven baseline methods on the QM9 dataset after filtering every molecule from the test set if it has a Tanimoto similarity score greater than 0.8 to any molecule in the training set (also see Figure S4A). (D) The comparison results of AUPRC between MoTSE and eleven baseline methods on an unbalanced PCBA dataset with only 10% positive samples (also see Figure S4B).

For each dataset of QM9 and PCBA, we sequentially treated one task in the dataset as a target task and the others as the source tasks. MoTSE measured the task similarity based on the models trained on the QM9_1*k*_ and PCBA_1*k*_ datasets. We performed three repeated tests with different random seeds and reported the averaged R^2^ and AUPRC scores on the QM9 and PCBA datasets, respectively (see Figure 3A). We found that MoTSE can make accurate predictions and outperformed all the baseline methods. As mentioned previously, transfer learning can lead to negative transfer (i.e., the performance of a transfer learning method is worse than that of training from scratch) when the source task is not properly defined. We plotted the prediction performance of each task from eleven transfer learning methods versus that from the training from scratch method (see Figure 3B). We observed that MoTSE perfectly avoided the negative transfer problem on the QM9 and PCBA datasets, while all the baseline methods suffered from this problem to varying degrees.

Next, we benchmarked MoTSE in more challenging scenarios. For the QM9 dataset, we filtered the test set of QM9_1*k*_ by excluding every molecule from the test set if it had a Tanimoto similarity score greater than 0.8 to any molecule in the training set (denoted by QM9_*filtered*_). For the PCBA dataset, we generated an unbalanced dataset with only 10% positive samples (denoted by PCBA_*unbalanced*_). We found that MoTSE still consistently outperformed baseline methods (see Figures 3C and 3D), and overcame negative transfer on all the tasks of these two representative challenging test cases (see Figure S4).

**Figure 4:**
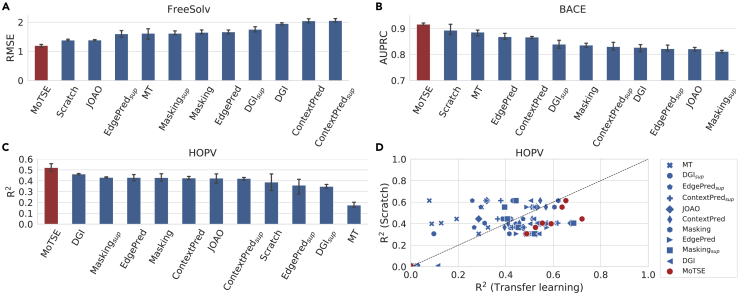
The prediction performance of MoTSE and baseline methods on the FreeSolv, BACE, and HOPV datasets (A) The comparison results between MoTSE and eleven baseline methods on the FreeSolv dataset, measured in terms of root-mean-square-error (RMSE). (B) The comparison results between MoTSE and eleven baseline methods on the BACE dataset, measured in terms of AUPRC. (C) The comparison results between MoTSE and eleven baseline methods on the HOPV dataset, measured in terms of R^2^. (D) The prediction performance of eleven transfer learning methods versus that of the Scratch method on the HOPV dataset, measured in terms of R^2^.

We also evaluated our method in more practical scenarios in which the source tasks and the target tasks were from different domains. More specifically, we first employed the FreeSolv dataset (Mobley and Guthrie, 2014), which produced a regression task measuring the solubility of 614 molecules. We derived the task similarity using the QM9_1*k*_ and FreeSolv datasets and used the tasks from the QM9_10*k*_ dataset as the source tasks. We employed MoTSE to enhance the transfer learning process and made a comparison with baseline methods. As shown in Figure 4A, MoTSE achieved better performance in comparison with baseline methods. Then we tested MoTSE on the BACE dataset (Subramanian et al., 2016), which measured whether each of 1513 molecules can act as an inhibitor of human β-secretase 1 (BACE-1). We first derived the task similarity using the PCBA_1*k*_ and BACE datasets, and then used the tasks from the PCBA_10*k*_ dataset as the source tasks. The comparison results between MoTSE and the baseline methods are shown in Figure 4B, which showed that our method still outperformed baseline methods. These results indicated that MoTSE can still accurately model the underlying similarity between molecular property prediction tasks even for the properties from different domains.

To further evaluate the ability of MoTSE in enhancing the prediction of molecular properties on extremely small datasets, we also accessed its performance on the HOPV dataset (Lopez et al., 2016), which contained only 350 molecules and measured eight quantum chemical properties. Here, we employed the tasks in the QM9_10*k*_ dataset as the source ones and used MoTSE to select the source task for each target task in the HOPV dataset. In comparison with baseline methods, MoTSE made more accurate predictions on the HOPV dataset and also achieved better results in addressing the negative transfer problem (see Figures 4C and 4D).

We also conducted additional tests to investigate the impact of the sizes of target and source datasets on the prediction performance of MoTSE (see Figures S6). Our analyses showed that the prediction performance of MoTSE was improved with the increase of the sizes of source and target datasets and MoTSE consistently outperformed Scratch, which demonstrated the robustness of MoTSE to the sizes of source and target datasets. Note that, MoTSE can still offer performance gain even when the source datasets only contain equal or fewer data samples than the target dataset (see Figures S6C and S6D). Based on these observations, we empirically recommended applying MoTSE on those target datasets with relatively limited data samples (e.g., less than 3,000) and employing source datasets that contain more data samples than target datasets, as MoTSE can achieve relatively larger performance gain under these conditions. Furthermore, we sought to define a proper threshold value of the similarity between the source task and target task that can effectively enable MoTSE to guide the transfer learning process. We first plotted the similarity between source and the target tasks versus the performance improvement on the QM9 and PCBA datasets, respectively. As shown in Figure S7, MoTSE achieved better prediction performance when the similarity between the source and target tasks was larger than 0.7.

### The task similarity estimated by MoTSE is generalizable across models with different architectures and datasets with different distributions

We next sought to explore whether the similarity estimated by MoTSE was generalizable across models with different architectures and datasets with different distributions, that is, whether the task similarity derived from MoTSE equipped with a certain model or on a certain dataset was generalizable to enhance the learning of other model architectures or datasets with different data distributions.

**Figure 5:**
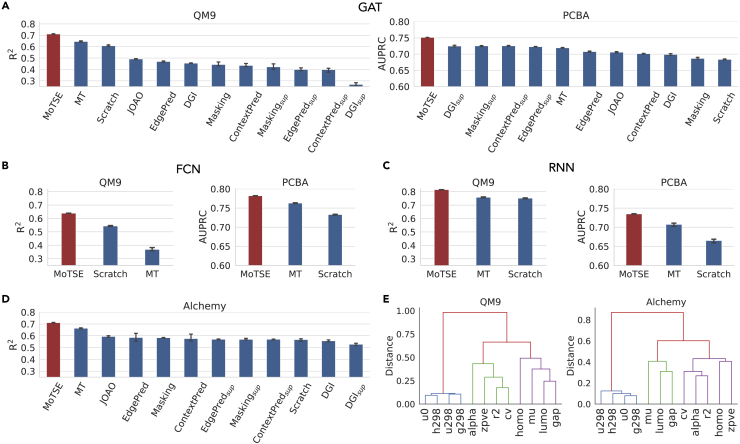
The task similarity derived from MoTSE is generalizable across models with different architectures and datasets with different data distributions (A-C) The comparison results between MoTSE and baseline methods on the QM9 and PCBA datasets (measured in terms of R^2^ and AUPRC, respectively), using the graph attention network (GAT), fully connected network (FCN), and recurrent neural network (RNN) models, respectively. (D) The comparison results between MoTSE and baseline methods on the Alchemy dataset, measured in terms of R^2^. (E) The similarity trees constructed based on the task similarity estimated by MoTSE on the QM9 and Alchemy datasets, respectively.

We first considered three models with different architectures in the tests, including a graph attention network (denoted as GAT) (Veličković et al., 2017), an ECFP (i.e., extended connectivity fingerprint) (Rogers and Hahn, 2010) based fully connected network (denoted as FCN) and a SMILES (i.e., simplified molecular input line entry specification) (Weininger, 1988) based recurrent neural network (denoted as RNN) (more details about these three types of models can be found in STAR Methods). Then, with the guidance of the similarity estimated by MoTSE equipped with the GCN model, we evaluated the transfer learning performance of the above three types of models on the QM9 and PCBA datasets and made comparisons with the baseline methods. Here we omitted the results of the nine self-supervised learning strategies on the FCN and RNN models, as they were particularly designed for GNNs and cannot be easily generalized to the FCN and RNN models. We observed that MoTSE consistently achieved significant improvement on the QM9 and PCBA datasets using different model architectures in comparison with all the baseline methods (see Figures 5A-5C). Moreover, we constructed similarity trees of tasks in the QM9 dataset using the hierarchical agglomerative clustering algorithm (Jain et al., 1999) according to the task similarity estimated based on GCN and GAT, respectively (see Figure S8). The similarity trees were highly consistent with each other. These results indicated that the task similarity estimated by MoTSE was generalizable across different model architectures.

Next, to evaluate the generalizability of the similarity estimated by MoTSE across datasets with different data distributions, we employed the Alchemy dataset (Chen et al., 2019), which shared the same tasks but had a different data distribution compared with the QM9 dataset, that is, the QM9 dataset contained molecules comprising up to nine non-hydrogen atoms while the molecules in the Alchemy dataset consisted of nine to fourteen non-hydrogen atoms. We first preprocessed the Alchemy dataset and created Alchemy_10*k*_ and Alchemy_1*k*_ following the preprocessing process shown in Figure S3. Then, for each source task, we pre-trained the models on the Alchemy_10*k*_ dataset. Next, for each target task in the Alchemy_1*k*_ dataset, we selected the source task from the Alchemy_10*k*_ dataset according to the task similarity estimated based on the QM9_1*k*_ dataset and fine-tuned on the Alchemy_1*k*_ dataset. We found that MoTSE still outperformed the baseline methods in this case (see Figure 5D). Moreover, we constructed the similarity trees according to the similarity estimated by MoTSE on the QM9 and Alchemy datasets, respectively. We observed that the structures of the derived similarity trees were highly consistent with each other (see Figure 5E).

These results demonstrated that the task similarity derived from MoTSE was generalizable across models with different architectures and datasets with different data distributions, which indicated that MoTSE can capture the model and dataset independent similarity between molecular property prediction tasks. Therefore, once the similarity between molecular property prediction tasks was estimated by MoTSE, it can be directly applied to enhance the learning of diverse model architectures and novel datasets in future studies.

### Task similarity derived from MoTSE reflects intrinsic relationships between physical chemistry properties

Next, we asked whether the task similarity derived from MoTSE was consistent with the intrinsic relationships between molecular properties. We constructed a dataset containing 10K molecules labeled with four well-studied physical chemistry tasks, including NHA (number of hydrogen acceptors contained in a molecule), NHD (number of hydrogen donors contained in a molecule), NOcount (number of nitrogen (N) and oxygen (O) atoms contained in a molecule), and NHOHCount (number of N and O atoms that are covalently bonded with hydrogens in a molecule) (see STAR Methods for more details of this dataset). Then we applied MoTSE to estimate the similarity between these tasks.

**Figure 6:**
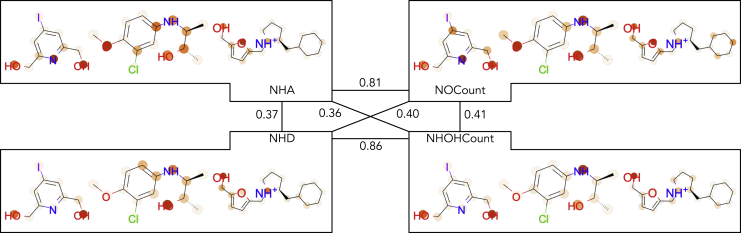
The similarity estimated by MoTSE between four physical chemistry tasks and the example molecules with importance scores assigned by the attribution method employed in MoTSE The numbers between tasks denote the task similarity derived from MoTSE. In the visualized molecules, darker colors represent higher importance scores. See the main text for the definitions of the four physical chemistry task.

From the chemical perspective, NHD is expected to be more similar to NHOHCount than NOCount, as only those N and O atoms with covalently bonded hydrogens can serve as hydrogen donors. NHA is expected to be more similar to NOCount than NHOHCount, as those N and O atoms both with or without covalently bonded hydrogens can be hydrogen acceptors. We observed that the task similarity derived from MoTSE was entirely consistent with these facts (see Figure 6).

Meanwhile, we visualized the importance scores of the atoms derived from the attribution method employed in MoTSE (see Figure 6). We found that MoTSE precisely assigned high importance scores to those target atoms related to the properties. For example, the N and O atoms were emphasized for the NOCount task, and the NH and OH atoms with hydrogen bonds were emphasized for the NHOHCount task. We also found that similar tasks tended to assign similar importance scores to the same atoms in molecules. For instance, NHD and NHOHCount both assigned higher importance scores to the N and O atoms with covalently bonded hydrogens. These observations interpreted how MoTSE estimated similarity between tasks and indicated that our method was able to capture the intrinsic similarity between tasks by exploiting the chemical concepts behind the corresponding molecular properties.

### Measuring and interpreting similarity between the tasks of estimating the bio-activities of molecules against cytochrome P450 isozymes

To further evaluate the ability of MoTSE in estimating and interpreting the similarity between molecular properties, we carried out a more challenging experiment, which included five tasks of predicting the bio-activities of small molecules against cytochrome P450 isozymes. The cytochrome P450 (CYP) family plays important roles in drug metabolism, especially for five isozymes—1A2, 2C9, 2C19, 2D6 and 3A4 (Williams et al., 2004; De Montellano, 2005). Here, we obtained the binary bio-activity labels between around 17K molecules and the above five CYP isozymes from the preprocessed ChEMBL dataset (Mayr et al., 2018). We then applied MoTSE to estimate the similarity of the tasks.

**Figure 7:**
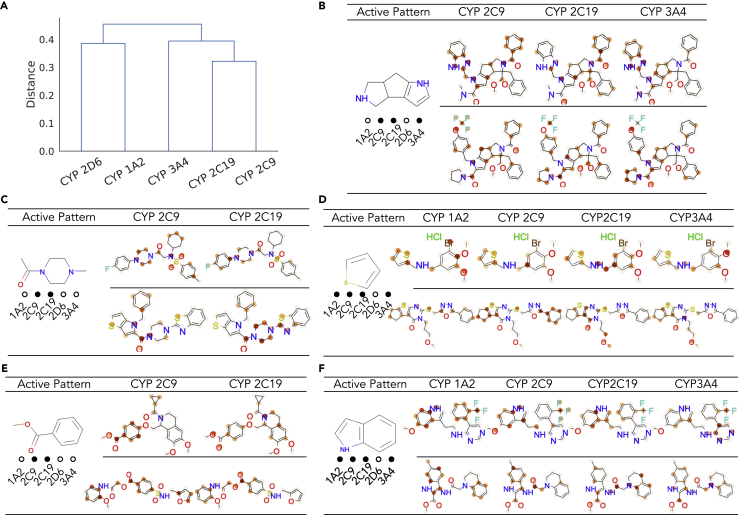
Measuring and interpreting the similarity between the tasks of estimating the bio-activities of molecules against cytochrome P450 isozymes (A) The task similarity tree constructed using the similarity estimated by MoTSE. (B-F) Five active patterns highlighted by our attribution method. The filled or hollow circles below a functional group represent whether the corresponding functional group is an active pattern for individual isozymes 1A2, 2C9, 2C19, 2D6 and 3A4 or not. The functional groups are shown on the left, while the molecules with the active patterns are shown on the right.

According to the similarity estimated by MoTSE, we first constructed a similarity tree using the hierarchical agglomerative clustering algorithm (Jain et al., 1999) (see Figure 7A). We observed that the similarity between CYP2C9 and CYP2C19 estimated by MoTSE was the highest among all pairs of CYP isozymes, which was consistent with the fact that CYP 2C9 and 2C19 genetically shared the most (91%) sequence homology (Attia et al., 2014). Meanwhile, we found that the structure of this tree was exactly the same as that derived by self-organizing maps (SOMs) (Schneider and Schneider, 2003; Selzer and Ertl, 2006) offered in previous research (Veith et al., 2009), in which the SOMs of individual isozymes were constructed based on the structural similarity of molecules and reflected the activity patterns (i.e., the scaffolds enriched in active or inactive molecules) of corresponding isozymes. According to this observation, we expected that MoTSE may capture the activity patterns of the CYP bioactivity prediction tasks and fully exploit such knowledge to estimate the similarity between these tasks.

To validate this hypothesis, we further visualized several molecules with the importance scores assigned by the attribution method employed in MoTSE. As shown in Figures 7B-7F, we found that similar tasks tended to share the same active patterns. For example, CYP 2C9 and CYP 2C19 shared the same five active patterns. In addition, the active patterns highlighted by our attribution method can be supported by previous research (Kho et al., 2006; Veith et al., 2009; Lee et al., 2017). For example, the substructure in Figure 7B was also previously considered as an active pattern of CYP 2C9, CYP 2C19 and CYP 3A4 by substructure searching (Veith et al., 2009) and fingerprint analysis (Lee et al., 2017).

The above results demonstrated that MoTSE can successfully extract task-related knowledge and thus accurately estimate the intrinsic similarity between the tasks (e.g., the similarity between active patterns and the genetic similarity between CYP isozymes). Therefore, MoTSE can potentially provide a novel perspective to help understand the mechanisms behind the bio-activities of small molecules.

### Conclusion

In this article, we present MoTSE, a computational method to efficiently estimate the similarity between molecular property prediction tasks. Specifically, we first pre-train a GNN to automatically capture task-related knowledge from the corresponding datasets. Then we employ the attribution method and the MRSA method to, respectively, extract both local and global knowledge contained in the pre-trained GNNs with the help of a probe dataset and project individual tasks as vectors into a unified latent task space. Finally, the similarity between the tasks can be measured by calculating the distances between the corresponding embedded vectors in the latent task space. The derived task similarity can be applied to design an accurate transfer learning strategy to enhance the prediction of molecular properties with limited data sizes. To ensure effective transfer learning, we empirically recommend applying MoTSE on the target datasets with limited data samples (e.g., less than 3,000) and employing the source datasets that contain more data samples than the target datasets. We also recommend selecting tasks with a similarity greater than 0.7 to the target task as the source tasks. In comparison with current transfer learning strategies, which attempt to leverage one proxy task to learn knowledge that can be generalized to molecular properties from different domains (i.e., self-supervised learning) or arbitrarily learn the target task and multiple source tasks simultaneously (i.e., multitask learning), our proposed transfer learning strategy offers a more reasonable and effective way to select a proper source task for each target task individually, thus fully taking advantage of the knowledge from the source task with sufficient data samples.

Comprehensive test results showed that the MoTSE-guided transfer learning strategy significantly outperformed the baseline learning strategies in predicting molecular properties and avoiding the negative transfer problem, especially on those datasets with limited data. MoTSE was also robust to different sizes of target and source datasets. Moreover, we validated that MoTSE achieved superior performance in the scenarios where the source and target tasks were from different domains. All these results demonstrated that MoTSE can be applied to molecular property prediction tasks from various scenarios. Therefore, MoTSE can provide a useful tool to fully exploit the increasing number of large-scale molecular property datasets to enhance the learning of properties with only limited training data, which is of great importance to accelerate the early stage of finding drug candidate molecules. In addition, we demonstrated that MoTSE can capture the intrinsic relationships between molecular properties and provide meaningful interpretability for the derived similarity, which can potentially help biologists/chemists understand the underlying mechanisms behind molecular properties.

### Limitations of the study

In our proposed learning strategy, we select the most similar source task to enhance the learning of one target task in a one-to-one transfer manner (i.e., transferring one source task to one target task). Although the test results have demonstrated the superior performance of such a strategy, there is still room for further explorations about improving the learning strategy. For example, we can design effective strategies to simultaneously take advantage of the top-*n* (n>1) similar tasks in the pre-training stage. In addition, a curriculum learning strategy can be designed by building effective learning paths (e.g., source task A → source task B → target task) based on the similarity derived from MoTSE. These points were not fully explored in our current work but will be interesting directions in future studies.

## STAR★Methods

### Key resources table

**Table:** 

REAGENT or RESOURCE	SOURCE	IDENTIFIER
**Software and algorithms**		

MoTSE	This study	https://github.com/lihan97/MoTSE
Python	Version 3.6.13	https://www.python.org/downloads/
PyTorch	Version 1.1.0	https://pytorch.org/
RDKit	Version 2018.09.3	https://www.rdkit.org/docs/Install.html
Deep Graph Library (DGL)	Version 0.4.2	https://www.dgl.ai/pages/start.html

**Other**		

QM9	(Ramakrishnan et al., 2014)	http://quantum-machine.org/datasets/
PCBA	(Ramsundar et al., 2015)	https://doi.org/10.48550/arXiv.1502.02072
Alchemy	(Chen et al., 2019)	https://www.dgl.ai/pages/start.html
FreeSolv	(Mobley and Guthrie, 2014)	https://alchemy.tencent.com/
BACE	(Subramanian et al., 2016)	https://doi.org/10.1021/acs.jcim.6b00290
HOPV	(Lopez et al., 2016)	https://doi.org/10.1038/sdata.2016.86

### Resource availability

#### Lead contact

Further information and requests for resources and reagents should be directed to and will be fulfilled by the lead contacts, Dan Zhao (zhaodan2018@tsinghua.edu.cn) and Jianyang Zeng (zengjy321@mail.tsinghua.edu.cn).

#### Materials availability

This study did not generate new unique reagents.

### Method details

#### Notation and problem setting

Suppose that we are given a set of molecular property prediction tasks $T=\left\{t_{1},t_{2},\ldots ,t_{N}\right\}$, where *N* stands for the total number of tasks involved. Accordingly, we have a set of datasets $D=\left\{D_{1},D_{2},\ldots ,D_{N}\right\}$, where $D_{i}=\left\{\left(x,y\right)\right\}$ stands for the dataset related to task $t_{i}$, and (x,y) represents a pair of molecule and its label for task $t_{i}$. We represent each molecule as a graph G=(V,E), where V stands for the set of nodes (i.e., heavy atoms) and E stands for the set of edges (i.e., covalent bonds). We use $u_{k}\in R^{N_{d}}$ to represent the initial features (e.g., atom type) of the *k*-th node in V, where $N_{d}$ stands for the dimension of node features.

Our goal mainly lies in the following 2-folds: (1) efficiently estimate the similarity between each pair of tasks in T; and (2) design an accurate transfer learning strategy based on the derived task similarity.

#### Key steps of MoTSE

##### Step 1: Pre-training the task-specific GNNs

The calculation of the similarity between tasks can be regarded as measuring the similarity of the intrinsic knowledge that needs to be learned from these tasks. Since deep learning models, especially the GNNs, have shown their superior capability of learning hidden knowledge and modeling various kinds of molecular properties (Gilmer et al., 2017; Li et al., 2019; Xiong et al., 2019), we adopt GNNs to capture such hidden knowledge contained in individual tasks. More specifically, for each task *t*, we pre-train a GNN model m=p(e(⋅)) using the corresponding dataset *D*, where e(⋅) acts as an GNN encoder to extract the latent feature representations of the molecule graphs and p(⋅) serves as a classifier or regressor (implemented through a multi-layer perceptron) to make prediction for *t*.

##### Step 2: Projecting tasks into task space

After pre-training the task-specific GNNs for individual tasks, the problem of measuring the similarity between tasks is converted into finding a way to quantitatively represent the knowledge enclosed in the pre-trained GNNs. MoTSE employs two knowledge extraction methods, including an attribution method and a molecular representation similarity analysis (MRSA) method, to derive the hidden knowledge from GNN models as represented vectors in a latent space.

Before elaborating on the task projection methods, we first define a probe dataset $D_{probe}=\left\{x_{1},x_{2},\ldots ,x_{N_{p}}\right\}$, which is a set of unlabeled molecules, where $N_{p}$ denotes the number of molecules. This probe dataset is shared across all tasks involved and acts as a proxy in the knowledge extraction process of each task to ensure that all the tasks can be projected into a unified latent space.

##### Attribution method

The attribution method is a way of interpreting deep learning models by assigning importance scores for individual input features to explain the prediction. Here, we use an attribution method to assign importance scores to individual atoms in each molecule from the probe dataset. The specific attribution method we use is Gradient∗Input (Shrikumar et al., 2016), which refers to a first-order Taylor approximation of how the output will change if a specific input feature is set to zero, thus indicating the importance of this input feature with respect to the output.

More formally, given the graph representation G for a molecule *x* from $D_{probe}$, the importance score $a_{k}$ of the *k*-th atom $u_{k}$ with respect to the task *t* can be computed as:

(Equation 1) $$a_{k}=\frac{1}{N_{d}}\overset{N_{d}}{\underset{f=1}{\sum }}u_{k,f}\times \frac{\partial \hat{y}}{\partial u_{k,f}},$$

where $u_{k,f}$ stands for the *f*-th element of the feature vector $u_{k}$, $N_{d}$ stands for the dimension of the input atom features, and $\hat{y}=m\left(G\right)$ stands for the prediction result of *x* for task *t* from the corresponding pre-trained GNN model *m*. Here, the importance score $a_{k}$ of the *k*-th atom is derived by averaging the importance scores of all dimensions of the atom features. After assigning the importance scores to individual atoms of molecule *x*, we obtain an attribution vector, denoted by $A=\left[a_{1},a_{2},\ldots ,a_{\left|V\right|}\right]$, where |V| stands for the number of atoms in *x*. By applying the above attribution method to every molecule in $D_{probe}$, we can derive the attribution vectors of all molecules in the probe dataset, denoted by $A=\left[A_{1},A_{2},\ldots ,A_{N_{p}}\right]$.

##### Molecular representation similarity analysis

As the attribution method scores each atom separately without considering the global information of molecules, we define such knowledge extracted by the above attribution method as local knowledge. Here, we also present a molecular representation similarity analysis (MRSA) method (Groen et al., 2018; Dwivedi and Roig, 2019) to extract the global knowledge learned from the pre-trained GNNs. In particular, for each task, we compute the pairwise correlations between the hidden molecule representations (i.e., the outputs of the encoders of the pre-trained GNNs) to depict the relationships between molecules in the latent molecular representation space.

More formally, for a task *t* and the encoder *e* from the corresponding pre-trained model, we first perform forward propagation for all molecules in $D_{probe}$ to generate their latent molecular representations $Z=\left[z_{1},z_{2},\ldots ,z_{N_{p}}\right]$, where $z_{m}$ stands for the latent molecular representation of molecule $x_{m}\in D_{probe}$. Then for each pair of molecular representations $z_{m}$ and $z_{n}$ (m≠n), we compute their correlation score $r_{m,n}$, that is,

(Equation 2) $$r_{m,n}=\rho \left(z_{m}\text{,}\;z_{n}\right),$$

where ρ(⋅) stands for the Pearson’s correlation coefficient. After that, we obtain a molecular representation correlation vector $R=\left[r_{1,2},\ldots ,r_{1,N_{p}},r_{2,3},\ldots ,r_{N_{p}-1,N_{p}}\right]$ as another vector representation of task *t*.

For individual tasks in T, MoTSE adopts the attribution method and the MRSA method mentioned above to extract both local and global task-related knowledge and projects them as vectors into two latent task spaces, denoted by $T_{A}$ and $T_{R}$, respectively.

##### Step 3: Estimating the task similarity

Once step 2 is completed, for each pair of tasks $t_{i},t_{j}\in T\left(i\neq j\right)$, their similarity can be computed in the latent task spaces $T_{A}$ and $T_{R}$:

(Equation 3) $$s_{i,j}^{A}=\frac{1}{N_{p}}\overset{N_{p}}{\underset{m=1}{\sum }}cosine\_sim\left(A_{m}^{i},A_{m}^{j}\right),$$

(Equation 4) $$s_{i,j}^{R}=cosine\_sim\left(R^{i},R^{j}\right),$$

where $s_{i,j}^{A}$ and $s_{i,j}^{R}$ represent the task similarity derived in $T_{A}$ and $T_{R}$, respectively, and cosine_sim(⋅) stands for the cosine similarity between two vectors.

The above two kinds of task similarities focus on different aspects to represent the hidden knowledge and are calculated under different assumptions. The attribution method mainly aims to extract local knowledge, and the assumption behind $s_{i,j}^{A}$ is that similar tasks should have similar importance scores for the same atoms in a molecule. On the other hand, MRSA mainly aims to extract the global knowledge, and $s_{i,j}^{R}$ measures the similarity on the basis that similar tasks should result in similar latent molecular representation spaces. To fully exploit the merits of both similarity estimation methods, we unify them into a more comprehensive formula:

(Equation 5) $$s_{i,j}=\left(1-\lambda \right)s_{i,j}^{A}+\lambda s_{i,j}^{R},$$

where λ stands for the weighting factor.

##### The MoTSE-guided transfer learning strategy

After deriving the similarity between pairs of tasks in T, for a target task $t_{i}\in T$, we can select the task $t_{j}$ (i≠j) with the highest similarity to $t_{i}$ as the source task. As such, we can fine-tune the model pre-trained on dataset $D_{j}$ to exploit the related knowledge from task $t_{j}$ and thus enhance the prediction of target task $t_{i}$.

#### Implementation of MoTSE

##### Training details

The full network architecture for the GCN model is illustrated in Figure S2. We adopted a graph convolutional network (GCN) (Kipf and Welling, 2016) implemented by the deep graph library (DGL) (Wang et al., 2019a) as the encoder to model the molecular graphs and a two-layer perceptron as the predictor to make prediction for molecular properties. More specifically, the GCN encoder had three 256-dimensional GCN layers and the predictor was a two-layer (512-256-1) fully connected network. We employed weighted sum pooling and max pooling as readout functions to produce the global feature representations of molecules and used a concatenation operation to combine these two derived feature representations as the final molecular feature representation. We employed ReLU as the activation function and set the dropout rate to zero. The pre-training and fine-tuning shared the same set of hyper-parameters. All the models were trained with the Pytorch framework (Paszke et al., 2017). The MSELoss and CrossEntropyLoss functions were employed to measure the mean-squared error and the cross entropy for the regression tasks and classification tasks, respectively. We used the Adam optimizer (Kingma and Ba, 2014) for gradient descent optimization with the following hyper-parameters: learning rate 1 × 10^−4^ and weight decay 1 × 10^−5^. All the models were trained for 200 epochs with early stopping, which aimed to terminate training when the validation accuracy had not been improved in the last 20 epochs. As MoTSE and baseline learning strategies are orthogonal to different model architectures, we did not tune the model configurations. The model and the training configurations of MoTSE were shared with baseline learning strategies for a fair comparison.

##### The probe dataset

In our tests, we constructed a probe dataset by randomly sampling 500 small molecules from the ZINC dataset (Sterling and Irwin, 2015). Although a larger probe dataset with carefully selected molecules may serve as a better proxy in the knowledge extraction process, intuitively, the empirical results demonstrated that 500 randomly selected molecules were sufficient to provide reliable estimations. More details about the effects of the randomness and the size of the probe dataset on the performance of MoTSE are provided in supplementary section 1.1.

##### Task similarity estimation

We empirically set the weighting factor λ to 0.7 in our computational experiments (see supplementary section 1.2 for more details).

#### Datasets and data processing

##### Datasets used to evaluate the prediction performance

We mainly used four representative datasets, including QM9 (Ramakrishnan et al., 2014), PCBA (Ramsundar et al., 2015), FreeSolv (Mobley and Guthrie, 2014), BACE (Subramanian et al., 2016), HOPV (Lopez et al., 2016) and Alchemy (Chen et al., 2019), to evaluate the effectiveness of our proposed method in molecular property prediction. QM9 is a dataset that provides twelve quantum chemical properties, such as geometric, energetic, electronic and thermodynamic properties of roughly 130K small molecules, associated with twelve regression tasks (Ramakrishnan et al., 2014). PCBA is a dataset consisting of biological activities of small molecules generated by high-throughput screening, associated with 128 classification tasks (Ramsundar et al., 2015). The FreeSolv dataset measures the hydration free energy of 642 small molecules in water from both experiments and alchemical free energy calculation (Mobley and Guthrie, 2014). The BACE dataset measures whether each of 1,513 molecules can act as an inhibitor of human β-secretase 1 (BACE-1) (Subramanian et al., 2016). HOPV is a dataset that provides eight quantum chemical properties containing 350 organic donor compounds (Lopez et al., 2016). The Alchemy dataset (Chen et al., 2019) shares the same tasks as the QM9 dataset but has different data distributions, that is, the QM9 dataset contains molecules comprising up to nine non-hydrogen atoms while the molecules in the Alchemy dataset consist of nine to fourteen non-hydrogen atoms.

We summarized the details of the tasks for the preprocessed QM9 and PCBA datasets, the FreeSolv dataset, the BACE dataset, the HOPV dataset and the Alchemy dataset in Table S1.

##### Dataset measuring physical chemistry properties

We constructed a dataset containing 10K molecules labeled with four physical properties, including counts of N and O atoms (NOCount), counts of NH and OH atoms (NHOHCount), number of H acceptors (NHA) and number of H donors (NHD). More specifically, We randomly sampled 10K molecules from the ZINC dataset (Sterling and Irwin, 2015) and derived the four properties from RDKit (Landrum, 2006).

##### Dataset measuring the bio-activities against cytochrome P450 isozymes

We also obtained a dataset that estimates the bio-activities of 17K molecules against five cytochrome P450 isozymes, including 1A2, 2C9, 2C19, 2D6 and 3A4, from a preprocessed ChEMBL dataset (Mayr et al., 2018).

#### Learning strategies

The task similarity derived from MoTSE was employed to guide the source task selection in transfer learning. More specifically, for each target task in QM9_1*k*_/PCBA_1*k*_, we selected *n* tasks with the top similarity scores as the source tasks from QM9_10*k*_/PCBA_10*k*_ according to the task similarity estimated by MoTSE, and took the best fine-tuning results as the final results. We set *n* to three and five for QM9 and PCBA datasets, respectively. The effect on the choice of *n* is provided in supplementary section 1.2.

To benchmark our proposed transfer learning strategy, we employed various previously defined transfer learning strategies, which mainly differed in the ways of defining source tasks and leveraging the knowledge from source tasks. More specifically, we employed multitask learning (denoted as MT), which learned the target task and all the available source tasks simultaneously, five self-supervised learning methods, including Masking (Hu et al., 2020), EdgePred (Hamilton et al., 2017), ContextPred (Hu et al., 2020), DeepGraphInfomax (Velickovic et al., 2019) (denoted as DGI) and JOAO (You et al., 2021), which first leveraged different proxy tasks to learn general knowledge from a large-scale unlabeled dataset and then fine-tuned the pre-trained model on the target dataset (here we used the ZINC dataset (Sterling and Irwin, 2015) with two million molecules to pre-train the self-supervised learning methods), and another four self-supervised learning methods, including EdgePred_*sup*_ (Hu et al., 2020), Masking_*sup*_ (Hu et al., 2020), ContextPred_*sup*_ (Hu et al., 2020) and DGI_*sup*_ (Hu et al., 2020), which first pre-trained the models using self-supervised strategies, then further pre-trained them on a preprocessed ChEMBL dataset (Mayr et al., 2018) by learning to predict bio-activities in a supervised fashion and finally fine-tuned the pre-trained models on target datasets. Moreover, we introduced the training from scratch scheme (denoted as Scratch) as a baseline method, which directly trained the model on the dataset of the target task and did not exploit any extra knowledge in the learning process.

Note that, our method is orthogonal to different GNN architectures. Here, for a fair comparison, we implemented all the learning strategies on the basis of GCNs if not specially specified, and we also used the same set of hyper-parameters for each method.

#### Model configurations

To evaluate whether the similarity estimated using GCNs can be generalized to guide the source task selection of other model architectures, we also considered other models, including graph attention networks (GATs) (Veličković et al., 2017), fully-connected networks (FCNs) and recurrent neural networks (RNNs) in our tests. The details of the models are provided below.

• GAT: GAT is a kind of graph neural network that employs the attention mechanism when performing message passing over nodes. We constructed a GAT model with three 256-dimensional GAT layers followed by a two-layer (512-256-1) fully connected network with the ReLU activation function (Agarap, 2018) for molecular representation extraction and property prediction.
• FCN: We built a five-layer (2048-1024-512-256-128-1) fully connected network with the ReLU activation function (Agarap, 2018) that took the ECFP (extended connectivity fingerprints) (Rogers and Hahn, 2010) representations of molecules as input to make molecular property prediction.
• RNN: RNN is a deep learning model particularly designed for processing sequential data, which has been proven to be effective in making molecular property prediction with SMILES (simplified molecular input line entry specification) representations (Weininger, 1988; Goh et al., 2018; Arús-Pous et al., 2019). Here, we employed a three-layer 128-dimensional LSTM (Hochreiter and Schmidhuber, 1997) (a classical variant of RNN) to encode SMILES representations of molecules into 64-dimensional latent vectors and a two-layer (64-32-1) fully connected neural network with the ReLU activation function (Agarap, 2018) to make predictions.

ECFP is a representation of 2D binary fingerprints (i.e., a series of bits) which can dynamically index the presence or absence of particular substructures of molecules. SMILES is a string-based molecular representation for describing molecular structures using short ASCII strings. Figure S1 gives an example for the ECFP and SMILES representations. In our computational experiments, we used DeepChem (Ramsundar et al., 2019) to calculate a 2048-bit ECFP for each molecule. For the SMILES string, we used one-hot vectors to encode the unique characters.

## Data Availability

• This paper analyzes existing, publicly available data. These accession numbers for the datasets are listed in the key resources table. • The source code and datasets of MoTSE can be found at https://github.com/lihan97/MoTSE. • Any additional information required to reanalyze the data reported in this paper is available from the lead contact upon request.

## Supplemental information

Document S1. Figures S1–S12, Tables S1–S5 and Note S1
