# Supplementary material for: Improving molecular property prediction through a task similarity enhanced transfer learning strategy
Source: iScience. 2022 Sep 30;25(10):105231. doi: 10.1016/j.isci.2022.105231 (PMC9579493; doi:10.1016/j.isci.2022.105231)
Supplement: Document S1. Figures S1–S12, Tables S1–S5 and Note S1 [file mmc1.pdf]

iScience, Volume *25*

Supplemental information

Improving molecular property prediction through a task similarity enhanced transfer learning strategy

Han Li, Xinyi Zhao, Shuya Li, Fangping Wan, Dan Zhao, and Jianyang Zeng

S1 Supplementary Notes

S1.1 Effect of the number of selected source tasks

To assess the effect of the number of selected source tasks (denoted by n) on our proposed transfer learning strategy, we plotted the prediction performance versus different values of n in Figure S9. We found that the prediction performance gradually improved with the increase of the value of n until the best performance that MoTSE can achieve (denoted by MoTSE*) was reached. To balance the accuracy and efficiency, we set n to three and five for the QM9 and PCBA datasets, respectively. We also introduced a baseline method that randomly selected source tasks for target tasks (denoted by Random) to make comparison with our proposed transfer learning strategy which exploited MoTSE to guide the source task selection.

S1.2 Effect of the Value of λ

We use λ to adjust the weights of the extracted local and global knowledge in the similarity estimation process. To evaluate the influence of the value of λ on the model performance, we plotted the prediction performance versus different values of λ in Figure S11a. We observed that MoTSE was robust to different values of λ ranging from 0.3 to 0.7. We set λ to 0.7 in our computational experiments.

S1.3 Effect of the Randomness of the Probe Dataset

The probe dataset is an important part of our MoTSE framework, which is shared across all tasks and acted as a proxy in the process of projecting each task into the unified latent task space, as described in the Method section of the main text. To evaluate the effect of different probe datasets, we first randomly sampled three probe datasets from the ZINC dataset and used MoTSE to estimate the similarity between tasks on the QM9$_{10k}$ and PCBA$_{10k}$ datasets using the three probe datasets, respectively. Then we measured the Pearson's and Spearman's correlations between the similarity values estimated using three different probe datasets for individual tasks. The average of Pearson's and Spearman's correlations across all tasks on the QM9 dataset were 0.999 and 0.977, respectively. The average of Pearson's and Spearman's correlations across all tasks on the PCBA dataset were 0.996 and 0.886, respectively. Such high correlation results indicated that MoTSE was robust to different probe datasets.

S1.4 Effect of the Size of Probe Dataset

We also investigated the effect of the size of the probe dataset on the prediction performance of MoTSE. We plotted the prediction performance of MoTSE versus different sizes of the probe datasets (see Figure S11b). From the results, the prediction performance kept relatively stable at high accuracy scores when the sizes of the probe dataset were larger than 300. We set the size of the probe dataset to 500 in our computational experiments.

S1.5 Ablation study

As we discussed in the main manuscript, MoTSE employed the attribution and MRSA methods to capture the local and global knowledge of molecular property prediction method, respectively. We have conducted an ablation study on MoTSE to investigate the effect on the prediction performance if we only used the attribution method (denoted by MoTSE$_{local}$) or the MRSA method (denoted by MoTSE$_{global}$) for the

task similarity estimation. As shown in Figure S10, MoTSE outperformed MoTSE$_{local}$ and MoTSE$_{glcoal}$ on both QM9 and PCBA datasets, indicating that MoTSE provided more accurate similarity estimations in comparison with MoTSE$_{local}$ and MoTSE$_{glcoal}$. Thus, MoTSE$_{local}$ and MoTSE$_{glcoal}$ are complementary to each other, and each of them contributes to the similarity estimation.

S1.6 Generalize to multi-to-one transfer learning

MoTSE performs transfer learning in a one-to-one fashion (i.e., transfer one source task to one target task). We also conducted new tests to evaluate whether a multi-to-one version of MoTSE (denoted by MTO) could provide better prediction performance. More specifically, for each target task, we first pre-trained a model by learning to predict the targets provided by its top-k $(k > 1)$ similar tasks in a multitask learning fashion and then finetuned the pre-trained model on the dataset of the target task. We set k to 2 and 3, resulting in two variations of MTO, denoted by MTO$_{k=2}$ and MTO$_{k=3}$, respectively. In addition, we also introduced the ideal versions of MTO$_{k=2}$ and MTO$_{k=3}$, denoted by MTO$^{*}_{k=2}$ and MTO$^{*}_{k=3}$, respectively, which always selected the k tasks that can achieve the best finetuning results as the source tasks in the pre-training stage. As shown in Figure S12, MoTSE outperformed all the MTO-based methods on both QM9 and PCBA datasets. Such results may be attributed to that the knowledge contained in the model pre-trained on multiple tasks was potentially more abstract and entangled which made the target task hard to take advantage of such knowledge in the finetuning process with very limited data. How to effectively exploit the related knowledge from multiple source tasks should be an interesting direction in future studies.

57 ## S2 Supplementary Figures and Tables

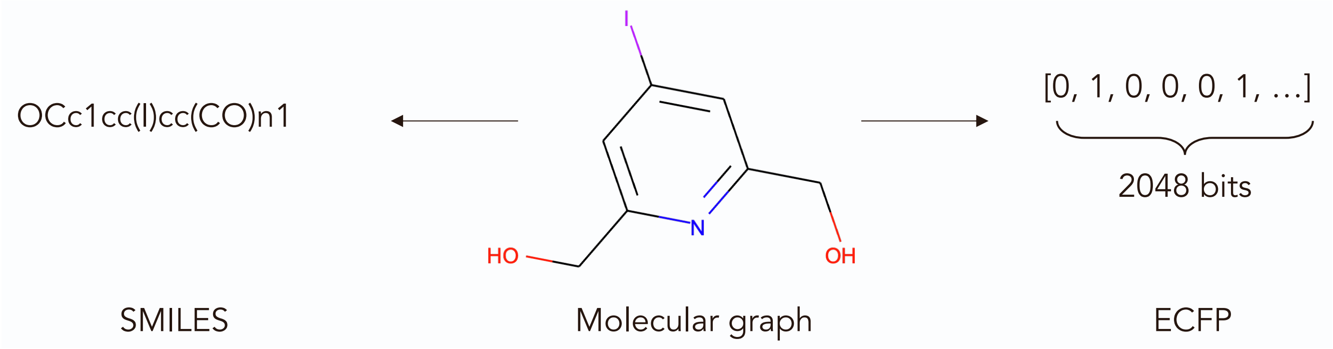

Figure S1: Three types of feature representations of an example molecule, related to Section 2.1 and STAR Methods. ECFP denotes the extended connectivity fingerprints, related to STAR Methods. SMILES denotes the simplified molecular input line entry specification.

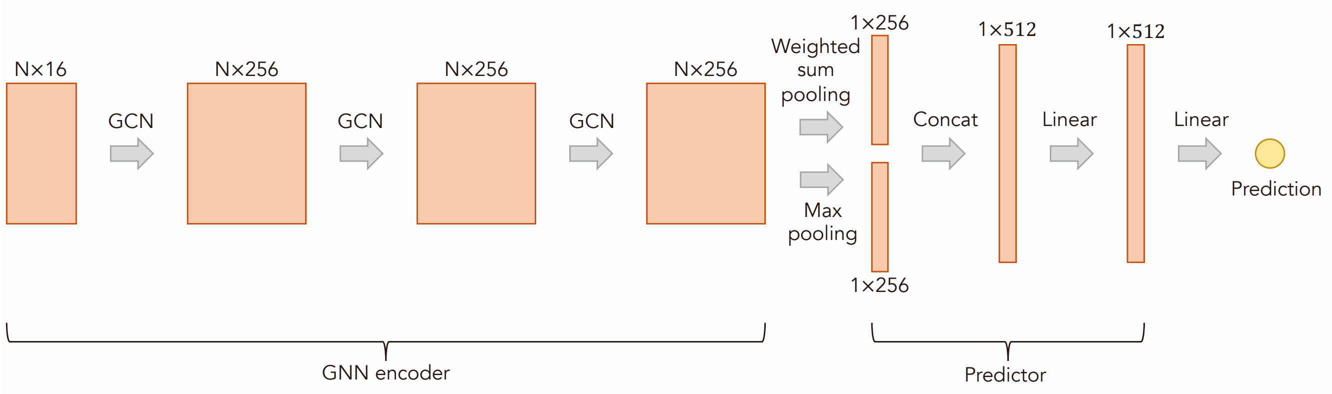

Figure S2: An illustrative diagram of the model architecture employed in our experiments, related to Section 2.1 and STAR Methods. N stands for the number of nodes in the input graph. The rectangles stand for the feature vectors and the numbers above the them stand for their dimensions. GCN stands for a graph convolutional network layer. Concat stands for the concatenation operation. Linear stands for a linear layer.

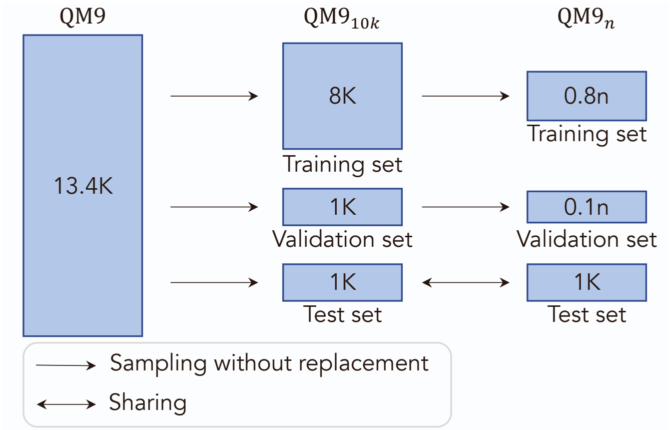

Figure S3: An illustrative diagram of the data generation process, related to Section 2.2. We sample QM9$_{10k}$ and QM9$_n$ from the QM9 dataset, where $n<10K$ stands for the size of the dataset. QM9$_{10k}$ and QM9$_n$ share the same test set for a fair comparison of prediction performance.

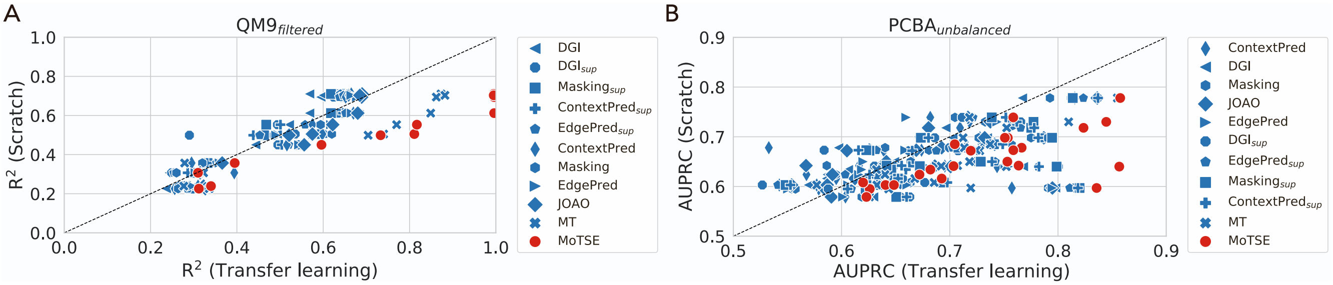

Figure S4: The prediction performance of seven transfer learning methods versus that of the Scratch method on the (A) QM9$_{filtered}$ and (B) PCBA$_{unbalanced}$ datasets, related to Figure 3.

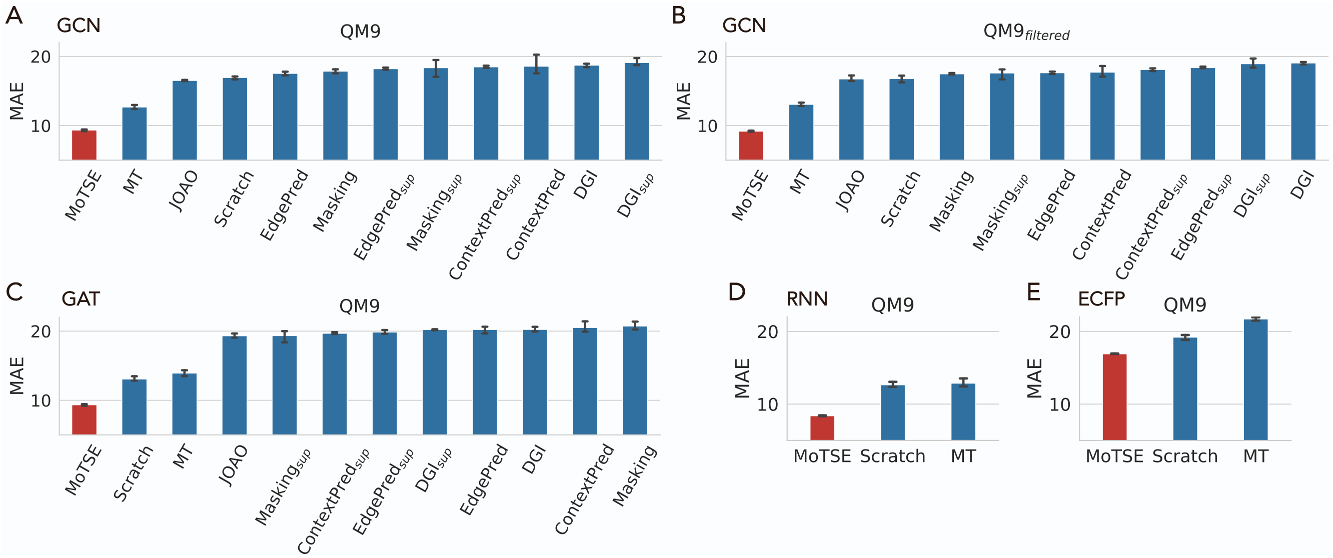

Figure S5: The mean absolute error (MAE) scores of MoTSE and baseline methods on the QM9 dataset, related to Figures 3 and 5. (A) The MAE scores on the QM9 dataset. (B) The MAE scores on the $QM9_{filtered}$ dataset. (C-E) The MAE scores of MoTSE equipped with (C) GAT, (D) RNN and (E) ECFP as backbones on the QM9 dataset.

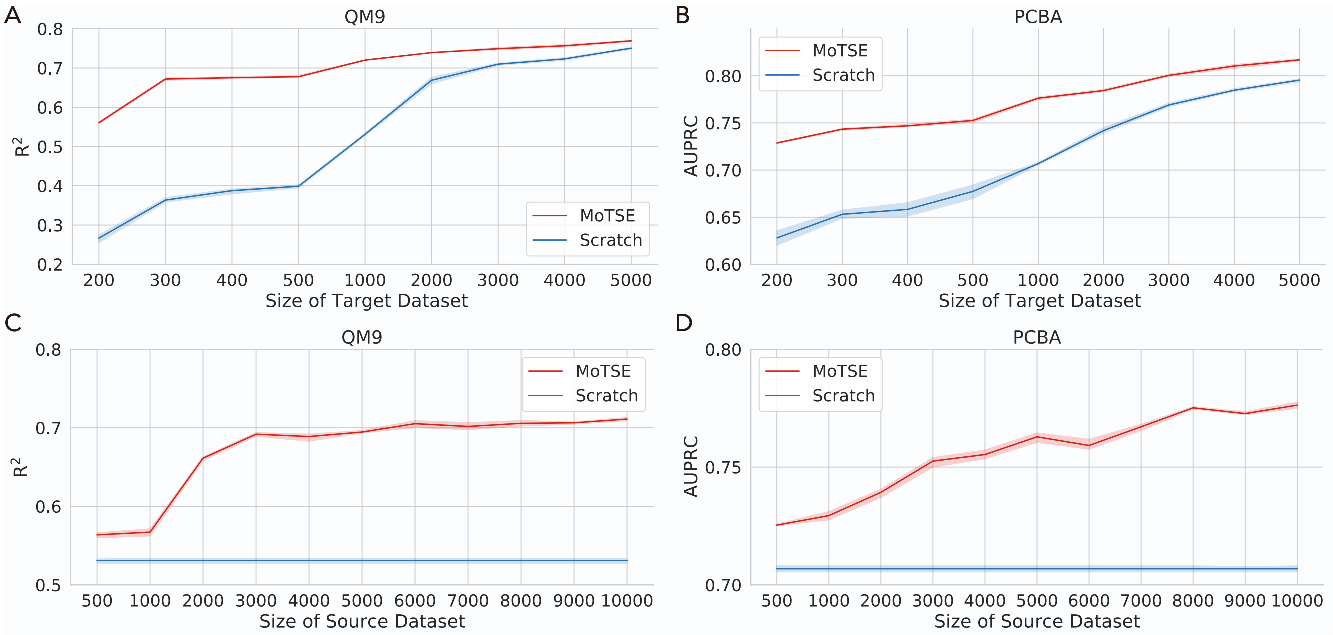

Figure S6: Impact of sizes of (A-B) target and (C-D) source datasets on the prediction performance of MoTSE, related to Section 2.2. The prediction performance of MoTSE and Scratch on (A) QM9 and (B) PCBA datasets, measured in terms of R^2 and AUPRC, given different sizes of target datasets (i.e., 500, 1000, 2000, 3000, 4000, 5000, 6000, 7000, 8000, 9000, 10000). The prediction performance of MoTSE and Scratch on (C) QM9 and (D) PCBA datasets, measured in terms of R^2 and AUPRC, given different sizes of source datasets (i.e., 200, 300, 400, 500, 1000, 2000, 3000, 4000, 5000).

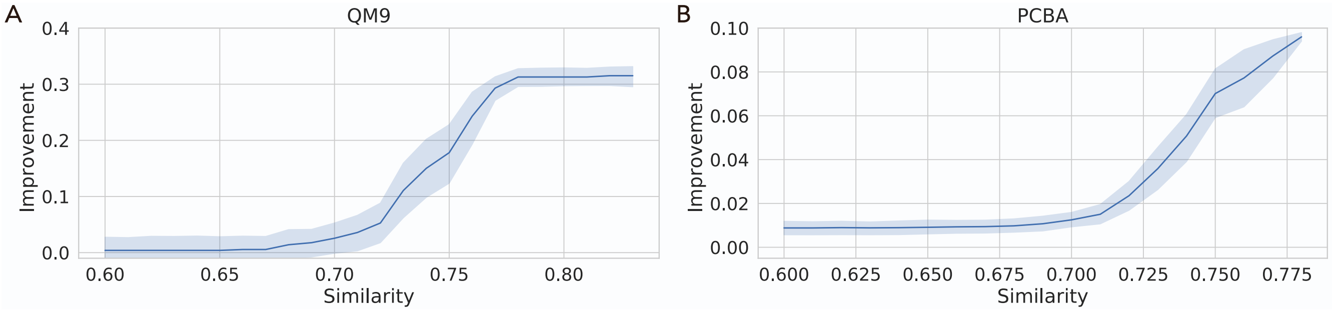

Figure S7: The similarity between the source and target tasks versus the performance improvement on the (A) QM9 and (B) PCBA datasets, related to Section 2.2.

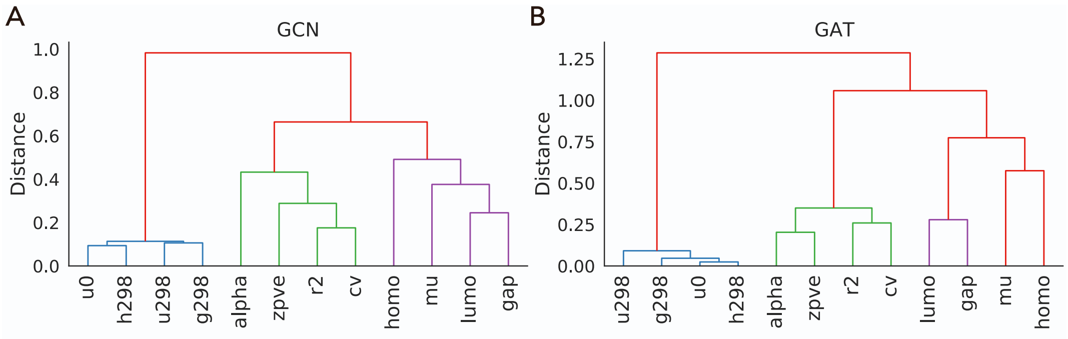

Figure S8: The similarity trees of tasks in the QM9 dataset constrcuted according to the task similarity estimated based on (A) GCN and (B) GAT, respectively, related to Section 2.3.

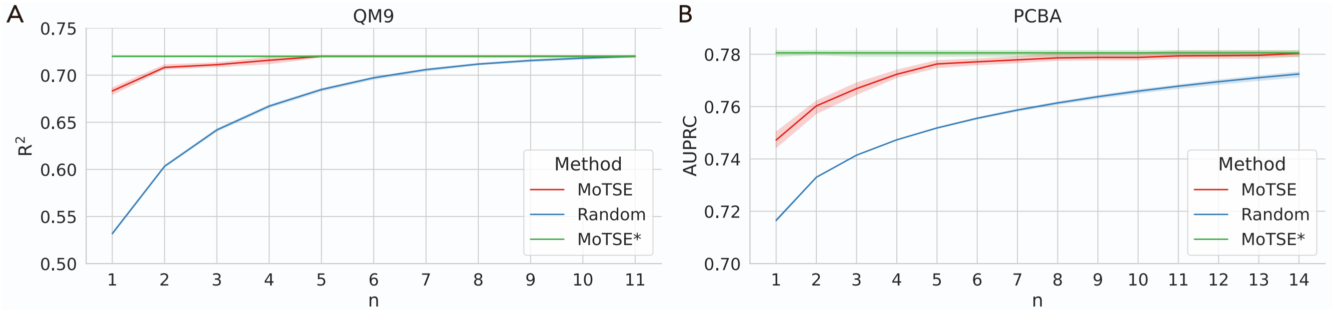

Figure S9: The prediction performance on the QM9 (A) and PCBA (B) datasets, measured in terms of R^2 and AUPRC versus different numbers of the selected source tasks (denoted by n), related to Supplementary Notes. Random denotes the results from the randomly selecting source tasks. MoTSE* denotes the best results that can be achieved by MoTSE.

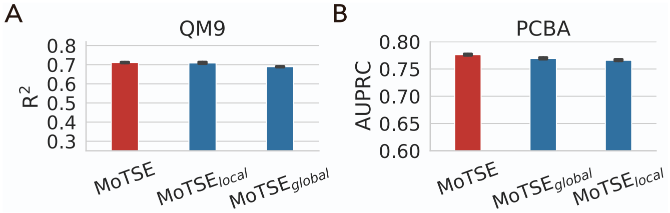

Figure S10: The prediction performance of MoTSE, MoTSE$_{local}$ and MoTSE$_{global}$ on the (A) QM9 and (B) PCBA datasets, measured in terms of R^2 and AUPRC, respectively, related to Supplementary Notes. MoTSE$_{local}$ and MoTSE$_{global}$ stand for the variation of MoTSE that only used the attribution method and MRSA method, respectively.

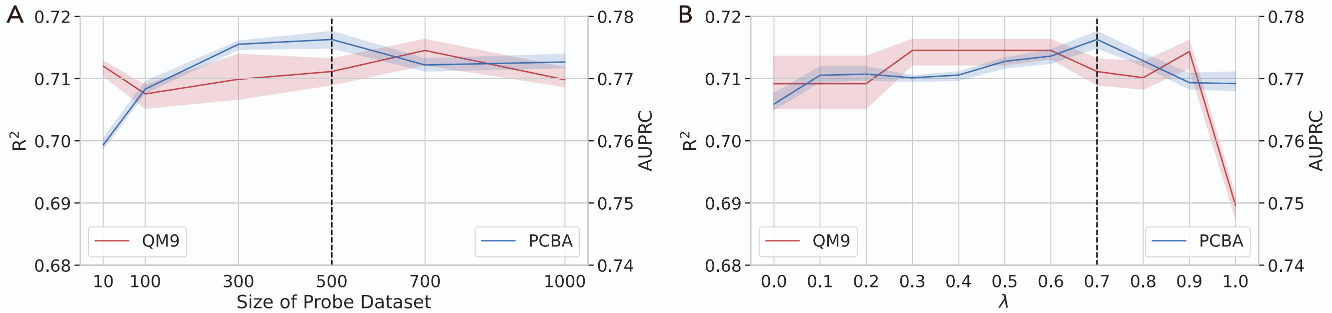

Figure S11: The prediction performance on the QM9 and PCBA datasets, measured in terms of R^2 and AUPRC with different values of λ (A) and different sizes of the probe datasets (B), related to STAR Methods and Supplementary Notes. The dashed line represents the final settings of λ and the size of the probe dataset.

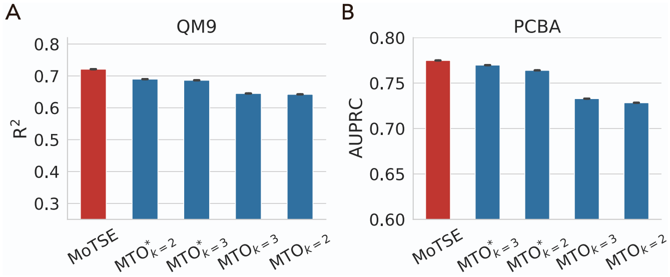

Figure S12: The performance comparisons between MoTSE and different multi-to-one transfer learning versions on the (A) QM9 and (B) PCBA datasets, measured in terms of R^2 and AUPRC, respectively, related to Supplementary Notes. MTO stands for the multi-to-one version of MoTSE, which selects the top-k ($k > 1$) similar tasks according to the similarity derived from MoTSE in the pre-training stage. MTO* stands for the ideal version of MTO, which always selects the k tasks that can achieve the best finetuning results as the source tasks.

Dataset	#Sample	Task	Description
$QM9_{10k}$ / $QM9_{1k}$	10K/1K	mu	dipole moment
		alpha	isotropic polarizability
		homo	energy of homo
		lumo	energy of lumo
		gap	gap between homo and lumo
		r2	gap between homo and lumo
		zpve	zero point vibrational energy
		u0	internal energy at 0 K
		u298	internal energy at 298.15 K
		h298	enthalpy at 298.15 K
		g298	free energy at 298.15 K
		cv	heat capacity at 298.15 K
$PCBA_{10k}$ / $PCBA_{1k}$	10K/1K	PCBA-1030	bio-activity against ALDH1A1
		PCBA-1458	bio-activity against SMN2
		PCBA-1460	bio-activity against K18
		PCBA-2546	bio-activity against VP16
		PCBA-2551	bio-activity against ROR
		PCBA-485297	bio-activity against Rab9
		PCBA-485313	bio-activity against NPC1
		PCBA-485364	bio-activity against TGR
		PCBA-504332	bio-activity against G9a
		PCBA-504333	bio-activity against BAZ2B
		PCBA-504339	bio-activity against JMJD2A
		PCBA-504444	bio-activity against Nrf2
		PCBA-504467	bio-activity against ELG1
		PCBA-588342	bio-activity against luciferase
		PCBA-624296	bio-activity against DNA re-replication
		PCBA-624297	bio-activity against DNA re-replication
		PCBA-624417	bio-activity against GLP-1
		PCBA-651965	bio-activity against ClpP
		PCBA-652104	bio-activity against TDP-43
		PCBA-686970	bio-activity against HT-1080-NT
		PCBA-686978	bio-activity against DT40-hTDP1
		PCBA-686979	bio-activity against DT40-hTDP1
		PCBA-720504	bio-activity against Plk1 PBD
HOPV	350	HOMO	energy of HOMO
		LUMO	energy of LUMO
		electrochemical_gap	minimal energy to create an electron hole pair in a semiconductor
		optical_gap	exciton energy which determines onset of vertical interband transitions
		PCE	power conversion efficiency
		V_OC	open-circuit voltage
		J_SC	short-circuit current density
		fill_factor	maximum power from a solar cell
BACE	1513	BACE	inhibitors of human β-secretase 1 (BACE-1)
FreeSolv	642	FreeSolv	hydration free energy in water

Table S1: Tasks and descriptions in the preprocessed QM9 and PCBA datasets, HOPV dataset, BACE dataset and FreeSolv dataset, related to Section 2 and STAR Methods. #Sample stands for the number of data samples for each tasks in the dataset.

Dataset		QM9	$QM9_{filtered}$	PCBA	$PCBA_{unbalanced}$	Alchemy	HOPV	FreeSolv	BACE
Metric		R^2	R^2	AUPRC	AUPRC	R^2	R^2	RMSE	AUPRC
Method	Scratch	$0.531_{(0.003)}$	$0.488_{(0.018)}$	$0.707_{(0.001)}$	$0.657_{(0.004)}$	$0.568_{(0.009)}$	$0.387_{(0.063)}$	$1.382_{(0.023)}$	$0.893_{(0.017)}$
	MT	$0.654_{(0.004)}$	$0.624_{(0.005)}$	$0.769_{(0.002)}$	$0.704_{(0.003)}$	$0.664_{(0.003)}$	$0.174_{(0.02)}$	$1.615_{(0.148)}$	$0.886_{(0.010)}$
	DGI	$0.499_{(0.045)}$	$0.439_{(0.002)}$	$0.699_{(0.001)}$	$0.662_{(0.004)}$	$0.557_{(0.006)}$	$0.462_{(0.006)}$	$1.950_{(0.034)}$	$0.827_{(0.012)}$
	EdgePred	$0.539_{(0.003)}$	$0.517_{(0.002)}$	$0.717_{(0.003)}$	$0.677_{(0.004)}$	$0.585_{(0.027)}$	$0.430_{(0.023)}$	$1.665_{(0.056)}$	$0.868_{(0.013)}$
	Masking	$0.538_{(0.004)}$	$0.509_{(0.004)}$	$0.711_{(0.003)}$	$0.665_{(0.002)}$	$0.583_{(0.002)}$	$0.429_{(0.028)}$	$1.647_{(0.061)}$	$0.836_{(0.006)}$
	ContextPred	$0.550_{(0.014)}$	$0.509_{(0.013)}$	$0.706_{(0.002)}$	$0.662_{(0.001)}$	$0.576_{(0.027)}$	$0.425_{(0.012)}$	$2.044_{(0.057)}$	$0.867_{(0.004)}$
	DGI_{sup}	$0.544_{(0.008)}$	$0.457_{(0.006)}$	$0.738_{(0.002)}$	$0.691_{(0.004)}$	$0.528_{(0.012)}$	$0.347_{(0.019)}$	$1.752_{(0.072)}$	$0.839_{(0.013)}$
	$EdgePred_{sup}$	$0.571_{(0.003)}$	$0.493_{(0.004)}$	$0.735_{(0.002)}$	$0.694_{(0.004)}$	$0.570_{(0.003)}$	$0.358_{(0.059)}$	$1.597_{(0.092)}$	$0.822_{(0.010)}$
	$Masking_{sup}$	$0.568_{(0.008)}$	$0.482_{(0.013)}$	$0.734_{(0.006)}$	$0.696_{(0.005)}$	$0.570_{(0.006)}$	$0.430_{(0.005)}$	$1.616_{(0.060)}$	$0.812_{(0.004)}$
	$ContextPred_{sup}$	$0.571_{(0.001)}$	$0.486_{(0.002)}$	$0.735_{(0.001)}$	$0.699_{(0.002)}$	$0.570_{(0.002)}$	$0.421_{(0.011)}$	$2.053_{(0.052)}$	$0.830_{(0.013)}$
	MoTSE	$0.711_{(0.002)}$	$0.691_{(0.002)}$	$0.776_{(0.001)}$	$0.733_{(0.001)}$	$0.711_{(0.002)}$	$0.521_{(0.029)}$	$1.198_{(0.042)}$	$0.916_{(0.003)}$

Table S2: The prediction performance of MoTSE and baseline methods on the QM9, $QM9_{filtered}$, PCBA, $PCBA_{filtered}$, Alchemy, HOPV, FreeSolv and BACE datasets, related to Figures 3 and 4.

Dataset		QM9	PCBA
Metric		R^2	AUPRC
Method	Scratch	$0.608_{(0.006)}$	$0.683_{(0.001)}$
	MT	$0.645_{(0.005)}$	$0.719_{(0.001)}$
	DGI	$0.455_{(0.002)}$	$0.699_{(0.002)}$
	EdgePred	$0.469_{(0.005)}$	$0.708_{(0.001)}$
	Masking	$0.443_{(0.016)}$	$0.687_{(0.002)}$
	ContextPred	$0.435_{(0.012)}$	$0.701_{(0.001)}$
	DGI$_{sup}$	$0.268_{(0.012)}$	$0.725_{(0.002)}$
	EdgePred$_{sup}$	$0.400_{(0.010)}$	$0.723_{(0.001)}$
	Masking$_{sup}$	$0.423_{(0.020)}$	$0.725_{(0.001)}$
	ContextPred$_{sup}$	$0.399_{(0.016)}$	$0.725_{(0.001)}$
	MoTSE	$0.711_{(0.002)}$	$0.751_{(0.0)}$

Table S3: The prediction performance of MoTSE and baseline methods equipped with graph attention network (GAT) on the QM9 and PCBA datasets, related to Figure 5A.

Dataset		QM9	PCBA
Metric		R^2	AUPRC
Method	Scratch	$0.543_{(0.003)}$	$0.733_{(0.001)}$
	MT	$0.369_{(0.010)}$	$0.763_{(0.001)}$
	MoTSE	$0.639_{(0.000)}$	$0.782_{(0.000)}$

Table S4: The prediction performance of MoTSE and baseline methods equipped with fully-connected network (FCN) on the QM9 and PCBA datasets, related to Figure 5B.

Dataset		QM9	PCBA
Metric		R^2	AUPRC
Method	Scratch	$0.751_{(0.003)}$	$0.665_{(0.005)}$
	MT	$0.758_{(0.003)}$	$0.707_{(0.004)}$
	MoTSE	$0.815_{(0.001)}$	$0.735_{(0.001)}$

Table S5: The prediction performance of MoTSE and baseline methods equipped with recurrent neural network (RNN) on the QM9 and PCBA datasets, related to Figure 5C.
